# Role of Gag in HIV Resistance to Protease Inhibitors

**DOI:** 10.3390/v2071411

**Published:** 2010-07-05

**Authors:** François Clavel, Fabrizio Mammano

**Affiliations:** 1 Inserm U941, Paris 75010, France; 2 Institut Universitaire d’Hématologie, Université Paris Diderot, Paris 75010, France; 3 Hôpital Saint Louis, AP-HP, Paris 75010, France; 4 Institut Pasteur, Unité Virus et Immunité, Paris 75015, France; 5 CNRS URA 3015, Paris 75015, France

**Keywords:** HIV-1, protease, resistance, mutations, Gag

## Abstract

Cleavage of Gag and Gag-Pol precursors by the viral protease is an essential step in the replication cycle of HIV. Protease inhibitors, which compete with natural cleavage sites, strongly impair viral infectivity and have proven to be highly valuable in the treatment of HIV-infected subjects. However, as with all other antiretroviral drugs, the clinical benefit of protease inhibitors can be compromised by resistance. One key feature of HIV resistance to protease inhibitors is that the mutations that promote resistance are not only located in the protease itself, but also in some of its natural substrates. The best documented resistance-associated substrate mutations are located in, or near, the cleavage sites in the NC/SP2/p6 region of Gag. These mutations improve interactions between the substrate and the mutated enzyme and correspondingly increase cleavage. Initially described as compensatory mutations able to partially correct the loss of viral fitness that results from protease mutations, changes in Gag are now recognized as being directly involved in resistance. Besides NC/SP2/p6 mutations, polymorphisms in other regions of Gag have been found to exert various effects on viral fitness and or resistance, but their importance deserves further evaluation.

## 1. Introduction

Protease inhibitors (PIs) are among the most active antiretroviral drugs currently used in the treatment of HIV infection. These compounds, which mimic the natural Gag and Gag-Pol substrates of the HIV protease, inhibit the proteolytic activity of the enzyme and exert a powerful inhibitory effect on HIV replication both *in vitro* and *in vivo*. In a large majority of treated patients, combinations of antiretroviral drugs that include PIs result in complete suppression of active HIV replication, and in remarkable recovery of immunodeficiency and reduction of AIDS-related mortality. However, as with all other antiretroviral drugs, failure by PIs to fully suppress HIV replication leads to the development of viral resistance. A unique feature of HIV resistance to PIs is the fact that resistance mutations not only arise in the protease itself – the direct target of the inhibitors – but also in some of the natural substrates of the protease – the Gag cleavage sites. We will review here the evidence that Gag cleavage site mutations are an important element of HIV resistance to PIs and discuss the mechanisms and implications of this phenomenon.

## 2. The HIV-1 protease and its natural substrates

The HIV protease is required to cleave the Gag and Gag-Pol polyproteins into their final functional protein products, leading to the assembly of a fully mature and infectious viral particle. The protease is encoded as part of the Gag-Pol polyprotein itself and becomes activated following a dimerization event that is only possible at sites where the concentration of Gag-Pol polyproteins is high, essentially at the site of virion assembly and budding. The protease domain cleaves itself out of the Gag-Pol polyprotein to constitute a symmetrically assembled homodimer, in which the substrate-binding site is a central, symmetrical cavity that is equally defined by each of the two subunits of the homodimer. Proteolytic cleavage of Gag and Gag-Pol polyproteins by the protease can be viewed as a switch from a configuration of the polyproteins that favors assembly and budding at the cell surface, to a configuration that promotes reassembly of a free mature capsid structure that can be released into the cytoplasm of the target cell, where it will deliver the fully functional replicative machinery of the virus. Proper assembly of this mature capsid structure requires that cleavage of the Gag and Gag-Pol polyproteins by the protease occur in an ordered and controlled manner [1–3].

**Figure 1:**
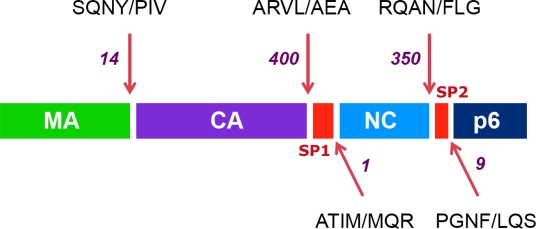
The ordered cleavage of the HIV Gag polyprotein precursor. The protein composition of the HIV Gag precursor is shown: colored boxes represent the internal structural proteins (matrix: MA, capsid: CA and nucleocapsid: NC), the protein p6 and the spacer peptides (SP1 and SP2). Arrows point to the position of cleavage sites. The sequences of the seven residues encompassing each cleavage site are indicated, with a slash representing the position of the scissile bond. Numbers indicate the decreases in the estimated rates of cleavage (from reference [3]), with the initial event between SP1 and NC set to a value of 1.

The order of cleavage of the Gag polyprotein is depicted in Figure 1. The first cleavage event separates the nucleocapsid protein (NC) from the capsid (CA) protein downstream of a 14-amino acid linker peptide termed SP1 (spacer peptide 1, formerly termed *p2*). Next, the CA protein is separated from the matrix (MA) protein, which remains associated with the virion membrane. This event that is almost simultaneous to the release of the C-terminal p6 Gag protein, downstream of another linker peptide located between NC and p6, termed SP2 (spacer peptide 2, formerly termed *p1*). Finally, the two linker peptides SP1 and SP2 are trimmed from the CA and NC proteins, respectively. The SP1 spacer peptide appears to play an important part in the overall maturation of Gag [4,5] and in the proper regulation of the ordered cleavage of Gag by the protease [3,6], however the role of SP2 remains unclear. An important factor in the ordered cleavage of Gag by the protease is the amino acid sequence of the cleavage site substrates [7]. These natural substrates are constituted of seven amino acids, whose position relative to the cleaved peptide bond are designated from N to C terminus: P4-P3-P2-P1 / P1’-P2’-P3’, with cleavage occurring between P1 and P1’ [7]. The amino acid sequence of the different cleavage site substrates within Gag strikingly differs from one site to another, some sites (most notably the SP1/NC site) being even remarkably polymorphic between HIV-1 clinical strains [8–12]. The difference in the amino acid sequence of these different substrates explains at least in part their differential rate of cleavage by the protease [2,7]. Interestingly, in spite of their marked sequence diversity, there is a strong similarity in the three-dimensional structure of these peptides in Gag [13,14], which explains why they all constitute strong and specific substrates for this enzyme, albeit with different cleavage rate efficiencies.

## 3. HIV resistance to PIs: protease mutations

HIV resistance to PIs is the consequence of accumulation of amino acid substitutions in the protease. During viral escape *in vitro* or *in vivo*, mutations accumulate gradually, leading to a progressive increase in the level of resistance [15,16]. Some mutations affect amino acids that are an integral part of the substrate binding domain of the enzyme: these mutations, often termed primary mutations, generally initiate the process of resistance evolution, and can differ from one PI to another. One of the most characteristic primary mutations is the substitution V82A, which is seen in most viruses having developed resistance to various PIs. Interestingly, this mutation modifies a strong point of contact between the enzyme and the inhibitors, but only one of the two valine residues at position 82 in the homodimeric protease is important for contact with the natural substrates [17]. Subsequently, secondary mutations are selected, which involve amino acids located away from the substrate-binding cavity. These secondary mutations are less drug-specific than primary mutations but are nonetheless critical for high-level resistance. Both primary and secondary mutations modify the shape and size of the substrate-binding cavity of the HIV protease, thereby reducing the affinity and the inhibitory potential of the inhibitors [18–20].

Overall, both primary and secondary resistance mutations appear to enlarge the substrate-binding cavity of the protease [18]. This enlargement seems to have more important consequences on the binding of the inhibitors, most of which are dependent upon a strong and tight interaction with the active site of the enzyme, than on the binding of the natural substrates of the protease in Gag and Gag-Pol. Indeed, Gag and Pol precursors interact less tightly with the enzyme [17–20], a property that is consistent with their ordered and regulated cleavage by the protease. With a few exceptions, strong and radical changes in inhibitor affinity cannot be achieved by single changes in the protease, and high level resistance requires subtle distortions of the substrate binding site that are both efficient in terms of resistance and tolerable for enzyme function.

## 4. HIV resistance to protease inhibitors and viral fitness

In spite of the subtle and gradual resistance evolution process described above, resistance to PIs is always a compromise between resistance and enzyme function. In fact, most PI-resistant viral strains display some extent of impairment of protease function and corresponding defects in replicative capacity or fitness [21–23]. Initial studies have suggested that only primary mutations had an impact on enzyme function and viral fitness, in view of their situation within the substrate binding cavity, and that secondary mutations were able to fully restore enzyme function and viral fitness [24]. In fact, most studies on clinical strains having developed resistance to protease inhibitors show that almost all viruses with high-level resistance display various degrees of fitness loss, even in the presence of multiple secondary mutations [21,22,25,26]. Interestingly, loss of protease function and loss of fitness can differ markedly from one resistant virus to another, with both differences in the levels of fitness impairment and differences in the nature of the cleavage defects in Gag or in Gag-Pol [21–23,26–28]. Therefore, secondary mutations in the protease may, in some instances, be quite effective in compensating for losses of fitness at early stages of protease resistance evolution, but strong pharmacological pressure by PIs, requiring further accumulation of resistance mutations and high-level resistance, almost always reduce viral fitness [25]. Resistance-associated loss of fitness is generally modest (two to 10-fold) in comparison with the increase in resistance (often >100-fold), explaining that natural selection by high concentrations of PIs would always favor highly resistant viruses even at the expense of some loss of fitness [21,25]. In this context, however, it was found that viral fitness and resistance were not only affected by mutations in the protease itself, but that both parameters were also strongly affected by mutations in Gag cleavage sites.

## 5. Cleavage site mutations as compensatory changes for resistance-associated loss of viral fitness

**Figure 2:**
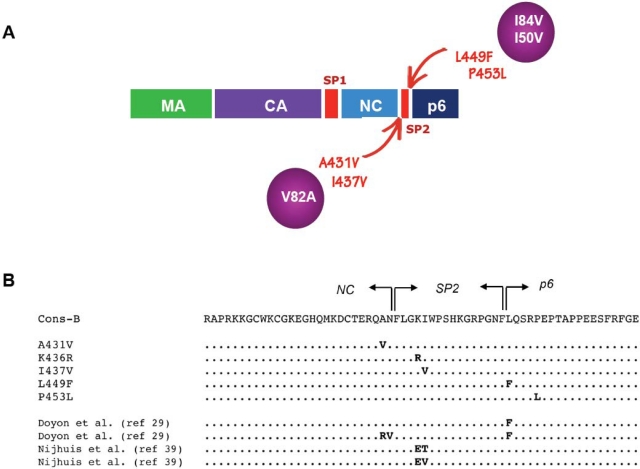
Frequently observed mutations at the NC/SP2/p6 cleavage sites. **A.** Gag mutations frequently observed in isolates from protease inhibitor-treated patients are shown in red, with arrows indicating their position in the Gag polyprotein. Protease mutations often found in association with these Gag mutations are shown in purple circles. **B.** Amino acid sequence of the subtype-B consensus, encompassing the NC/SP2/p6 cleavage sites in Gag. Arrows indicate the nucleocapsid (NC), SP2 and p6 domains. The first set of five sequences below the consensus illustrates Gag mutations frequently observed in virus isolates from treated patients. The following set of four sequences shows the position of Gag mutations obtained from *in vitro* studies with experimental protease inhibitors. Dots indicate amino acid identity to the subtype-B consensus.

Initial observations of mutations in Gag cleavage sites during selection for resistance to PIs were made by Doyon *et al.* [29] in viruses subjected to *in vitro* selection by experimental inhibitors BILA 1906 BS and BILA 2185 BS. The mutations were located within the two cleavage sites that define the SP2 peptide between NC and Gag p6 (Figure 2). In one experiment with BILA 1906 BS, an L to F substitution at position P1’ of the SP2/p6 cleavage site was observed, a mutation designated L449F when numbered according to the whole Gag polyprotein amino acid sequence. This mutation was found to emerge shortly after selection of three mutations in the protease, including the mutation I84V. In a second experiment with BILA 2185 BS, mutation L449F was again selected, but was shortly followed by emergence of dual mutations Q430R and A431V at positions P3 and P2, respectively, of the NC/SP2 site. Interestingly, the presence of these mutations had a clear effect on virus replication kinetics, but no changes in IC_50_ were observed.

Mutations in Gag cleavage site were also found in viruses from treated patients. In a series of samples from indinavir-treated subjects in whom treatment failed to control HIV replication, Zhang *et al*. [30] observed early emergence of mutation A431V in the NC/SP2 cleavage site in three patients, while substitution L449F in SP2/p6 was seen at a later time point in one patient. In another study involving 16 patients having failed treatment by ritonavir or saquinavir, Mammano *et al*. [28] observed the presence of the mutation A431V in NC/SP2 in two instances and mutation of L449F in SP2/p6 was seen in one case. Further study on the replicative capacity of recombinant proviral clones carrying either Gag-protease or protease alone from these three viruses revealed that the mutated Gag sequences were able to compensate at least partially for the loss of fitness conferred by the cognate mutated proteases. The rescue of virus replicative capacity was associated with, and supposedly due to, improved cleavage at the mutated site by resistant proteases. Thus, it became accepted that the role of Gag cleavage site mutations was essentially related to viral replicative capacity or fitness.

Following these initial observations, several studies pointed to the importance of Gag cleavage site mutations in the evolution of HIV resistance to PIs [9,31–36]. New mutations were observed, some of which are located in close proximity – although not within the canonical seven amino acid sequence – of the cleavage sites, in particular the mutation I437V downstream of the NC/SP2 site, and mutation P453L downstream of the SP2/p6 site [33,34]. Strong associations between some of these Gag mutations and specific protease resistance mutations were seen. Indeed, while Gag mutations A431V and I437V are almost constantly associated with protease resistance genotypes that include the mutation V82A, the Gag mutation L449F is most frequently seen in association with I84V in the protease [8,35]. It has even been suggested, but not clearly demonstrated, that emergence of the I84V mutation could actually be favored by the preexisting mutation L449F in Gag [8]. Mutation P453L, which can be seen in some primary HIV-1 sequences from treatment-naïve patients, can nonetheless emerge in association with the mutation I84V or with the amprenavir-specific mutation I50V [34]. These associations were validated and extended in a relatively large survey (500 patients) that compared Gag cleavage site sequences from treatment-naive and -experienced patients [37]. The most frequent therapy-associated mutations in the NC/SP2 cleavage site (A431V and I437V) were observed in association with protease mutations at positions 82 and 54, while leading mutations at the SP2/P6 cleavage site (L449F and P453L) were found in the context of protease mutations D30N or I84V. A detailed table of the individual frequency of association can be found in the original article [37]. Although some viruses from this and other studies (e.g., [31]) do not fit into this simple pattern, such a general association rule summarizes data from several reports. Tighter associations should not be expected, because of the multiple parameters that govern the emergence of mutations in PR and Gag, such as the specific inhibitor used, the order of exposure to different inhibitors, the duration of treatment and pre-existing Gag polymorphisms. Finally, there is no apparent mechanistic or evolutionary conflict between mutations affecting the NC/SP2 or SP2/p6 sites. Some data, however, suggest that the mutations A431V and I437V, both being favored in the context of the protease mutation V82A, are not seen on the same genomes and may be incompatible with each other [38].

## 6. Cleavage site mutations as resistance mutations

Besides the well documented effect of Gag cleavage site mutations on resistance-associated loss of viral fitness, evidence has been accumulating that these mutations could also directly affect HIV susceptibility to PIs in a manner that is independent of their effect on fitness. In their 1997 study, Zhang *et al*. [30] observed that the presence of the mutation A431V in NC/SP2 could improve the kinetics of viral replication not only in the absence of indinavir, but also in the presence of moderate concentrations of that drug. Similarly, Carron de la Carrière *et al*. [27] observed that the presence of the same A431V mutation strongly improved the selective advantage profile of some protease mutants in the presence of ritonavir, a parameter that reflects the ability of viral variants to outgrow wild-type virus according to the concentration of drug in the culture. In line with these earlier findings, Maguire *et al*. [34], studying the effect of Gag mutation P453L in viruses carrying the amprenavir-specific I50V protease mutation, again observed that beyond merely correcting viral fitness, the Gag mutation could also significantly increase the IC_50_ of amprenavir in these mutants. Finally, Prado *et al*. [39] reported that the Gag mutation L449F, in the context of amprenavir-selected mutations L10F/I84V, increased phenotypic resistance to all PI tested. In these four studies, however, the extent to which the increase in resistance was dependent or independent of the effect of Gag mutations on viral fitness was not clearly determined. This point was solved by two recent studies [40,41], which clearly demonstrated that Gag cleavage site mutations, independently of their role in viral fitness, should be considered as authentic PI resistance mutations.

**Figure 3:**
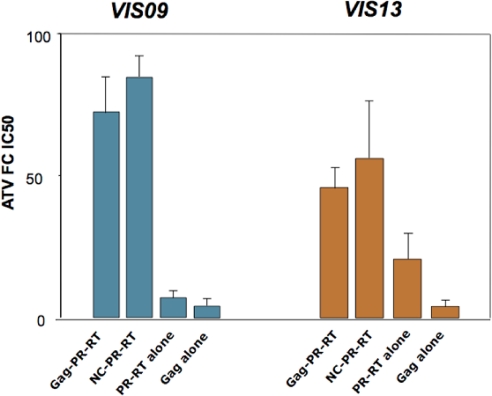
Effect of Gag on protease inhibitor resistance. Different combinations of Gag and Pol sequences from two protease inhibitor-experienced patients (VIS09 and VIS13) were used to replace the corresponding regions from an HIV molecular clone (NL4.3). The fold-increase in IC_50_ (as compared to wild type NL4.3 virus) for the protease inhibitor Atazanavir (ATV) is shown on the Y-axis. For both individuals, patient-derived Gag sequences (Gag-PR-RT, and NC-PR-RT) were required for high-level resistance, as the clones carrying only Pol from patient (PR-RT alone) displayed significantly lower resistance levels. In particular, the C-terminus of Gag (encompassing the NC/SP2/p6 cleavage sites) was sufficient to confer high-level resistance in the context of patient-derived Pol (NC-PR-RT). Primary Gag sequences from these two patients, expressed in the absence of protease mutations (Gag-alone), did not confer statistically significant resistance to ATV. Data are from the study in reference [38].

In a study by Nijhuis *et al*. [41], a laboratory strain of HIV-1 was subjected to selection through repeated cell culture passaging in the presence of increasing concentrations of an experimental PI, RO033-4649. Remarkably, viral variants selected by this process did not carry any resistance mutations in the protease. Instead, these variants exhibited mutations in the SP2 spacer peptide, K436E and I437T or V. Introduction of these mutations in combination in a wild-type reference proviral clone showed a clear, although moderate, increase in the IC_50_ of RO033-4649 and of other PIs, thereby demonstrating the direct impact of Gag mutations on resistance to a wide range of protease inhibitors, in the absence of protease mutations. The impact of Gag cleavage site mutations in PI resistance was further emphasized in a study by Dam *et al*. [40], examining the phenotype of different recombinant viral clones carrying different Gag or Gag-Pol segments from highly evolved viruses that had accumulated multiple PI resistance mutations in treated patients. All of these primary viral sequences also carried one or more of the characteristic mutations in the NC/SP2/p6 Gag cleavage sites as described in Figure 2. In this study, it was found that the association of mutated Gag sequences together with mutated protease sequences produced markedly higher IC_50_ levels and fitness values. This effect was fully recapitulated by a segment of the viral genome expressing the NC/SP2/p6 region of Gag (Figure 3). Strikingly, reversion of individual cleavage site mutations A431V or I437V in these viruses markedly reduced their level of resistance, but had only a marginal effect on fitness, strongly suggesting that these mutations were indeed essentially acting by themselves as authentic resistance mutations. In contrast, their benefit in terms of viral fitness appeared more complex, and while the mutations within cleavage site sequences such as A431V or I437V certainly exert a positive impact on fitness at early stages of resistance evolution, this effect may be diverted or confounded by further co-evolution and adaptation of as yet poorly defined determinants in the NC/SP2/p6 region.

## 7. Mechanisms of action of Gag cleavage site mutations

The mechanisms through which Gag cleavage site mutations can increase resistance and improve viral fitness are yet to be fully understood. These mechanisms are centered on three key questions.

**Figure 4:**
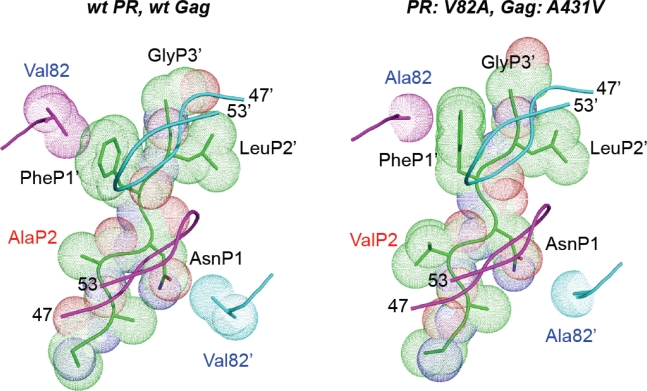
Resistance-associated modification of contacts between protease and Gag. Representation of the NC/SP2 cleavage site (the amino acid backbone and surface occupancy are illustrated) within the protease active site. Left: wild-type protease and Gag. Right: protease carrying a V82A resistance mutation and Gag harboring the A431V mutation. Both views are from above the flap region of protease (top views). The backbones of the flaps (residues 47 to 53) are illustrated as a light blue- and a violet ribbon above the surface of the cleavage site. The protease residues (Val and Ala for wild type and mutated protease, respectively) at position 82 and 82’ in the active site of the enzyme are shown. Replacement of Val82’ by the smaller Ala82’ in the resistant protease (bottom right of the right panel) results in the loss of contact with the Asn residue in position P1 (AsnP1) of the cleavage site. This can be partly compensated by the Gag mutation A431V, which creates a protrusion in position P2 of the cleavage site (ValP2 on the right panel, in red). This is expected to create a new contact with the protease active site (not shown), at a position that is not normally occupied by the substrate.

The first question, addressing how single mutations act locally on the interaction between the cleavage site substrate and the protease, has been partially answered by structural crystallographic studies of wild-type or mutated substrates in the presence of wild-type or mutated protease. In particular, it is well understood how the A431V mutation can improve cleavage of the NC/SP2 site by a protease bearing the V82A mutation (Figure 4) [17]. The smaller alanine residue at position 82 in the protease, compared to wild-type valine, opens a space between the enzyme and protease inhibitors, thereby decreasing affinity of the inhibitor for the enzyme and producing resistance. This change, however, also reduces to some extent the contact between the protease and the NC/SP2 substrate. As shown in Figure 4, the A431V mutation creates a protrusion of the NC/SP2 peptide in a space within the substrate-binding domain that is not naturally occupied by the substrate. Interestingly, this structural change does not involve any of the substrate residues that are in direct contact with the amino acid at position 82, but instead creates a different, alternative contact between the enzyme and substrate that compensates for the loss of contact resulting from the protease mutation. This model, following which substrate changes can fill gaps between substrate and mutated enzyme and thereby improve cleavage, provides a highly credible explanation for the effect of Gag cleavage site mutations on viral fitness and is likely to apply to other cleavage site mutations observed in PI-resistant viruses. It is corroborated by observations that cleavage sites carrying the characteristic mutations described earlier behave as better substrates for the protease *in vitro*, whether mutated or wild-type [29]. This improvement in cleavage cannot be selected for in the absence of PIs, most likely in view of the fact that cleavage at the NC/SP2/p6 sites needs to be fully balanced with cleavage at other sites, and that viruses with increased NC/SP2/p6 cleavage efficiency may not necessarily be advantaged in the absence of PIs and/or in the context of a wild-type protease.

The second question that needs to be answered is why most PI resistance-associated Gag mutations are clustered around the SP2 peptide. Coren *et al*. [42] have evaluated the replicative importance of cleavage at the NC/SP2 and at the SP2/p6 sites in viruses carrying mutations that reduced cleavage at these sites. Surprisingly, full obliteration of NC/SP2 cleavage in one of these mutant viruses did not affect HIV-1 replication. This finding strongly contrasts with the observations of Dam *et al*. [40], where it appeared that the extent of cleavage at this site, as measured by the amount of mature NC protein relative to incompletely cleaved NC-SP2 products in recombinant viruses carrying wild-type or A431V mutated NC/SP2 sequences, was proportional to virus infectivity, whether in the presence (a measure of resistance) or in the absence (a measure of fitness) of protease inhibitor lopinavir. Regarding the importance of improving cleavage at the SP2/p6 sites, the mutational studies by Coren *et al*. cited above suggest that cleavage at this site is critical for HIV infectivity. The extent to which mutations in SP2/p6, in SP2, or even in NC/SP2, could impact SP2/p6 cleavage and release of mature p6 protein is unclear. In the Nijhuis *et al*. study [41], where viruses carrying substitutions at positions 436 and 437 within SP2 were studied, these mutations appeared to decrease the amounts of an incompletely cleaved NC-SP2-p6 intermediate product, but their effect on the amounts of mature p6 protein was not seen.

The third unanswered question is to understand how improved cleavage by the protease can produce resistance, independently of changes in fitness. One possibility is that a better substrate will constitute a better competitor for protease binding in the presence of protease inhibitors. This may appear unlikely, especially in view of the fact that affinity of PIs for the HIV protease is several orders of magnitude greater (picomolar *versus* micromolar) than the affinity of Gag cleavage sites [43]. This potential balance, however, may be strongly affected by the local concentration of the competing partners: the concentration of Gag cleavage sites can be considered as being very high at the site of virion assembly, release and maturation, and the intracellular or intravirion concentration of PIs is largely unknown. An alternative model has been proposed, according to which Gag cleavage site mutations would improve the efficiency of ribosomal frameshift at the Gag-Pol junction, thus increasing the amounts of protease [44]. This model, however, remains controversial [45].

## 8. Role of other mutations or polymorphisms in Gag in HIV resistance to PIs

Most studies on the role of Gag in resistance to PIs have essentially focused on mutations affecting cleavage site sequences in the NC/SP2/p6 region of Gag. In their study establishing the role of cleavage site mutations in this region, Dam *et al*. [40] dismissed a potential role of changes in other Gag domains in resistance and/or fitness. Indeed, the phenotype of recombinants carrying only patient-derived NC/SP2/p6 domains from highly resistant patient-derived viruses did not differ from that of recombinants carrying whole patient-derived Gag sequences. Recent data by Parry *et al*. [46], however, also studying Gag sequences from highly resistant primary viruses, clearly demonstrated that domains in the matrix (MA) and capsid (CA) protein of HIV-1 were able to confer higher resistance and improved replicative capacity to viruses bearing mutations in the protease. Unlike the mechanisms discussed above, these changes do not directly target the MA/CA cleavage site. Instead, multiple polymorphisms are involved, which could improve fitness and resistance by improved MA/CA cleavage through modifications of the overall conformation of the Gag precursor. Two other studies have examined the impact of Gag mutations outside of cleavage sites on the fitness of PI-resistant viruses [47,48]. Both studies observe that changes in the NC/SP2/p6 region can significantly improve fitness of replication-impaired viruses carrying mutations in the protease. These phenomena, in line with the observations of Dam *et al*. on the fitness of viruses in which NC/SP2 mutations had been reverted, may again relate to increases in cleavage efficiency by overall conformational changes of this region of Gag.

## 9. Gag cleavage site mutations in a clinical context: frequency, kinetics of emergence, and impact on treatment response *in vivo*

Existing data on the frequency of cleavage site mutations in treatment-naïve and -experienced patients is limited. Recently Verheyen *et al*. [12] explored the prevalence of polymorphisms and treatment-associated mutations in the NC/SP2/P6 cleavage sites (431V, 436R, 437V, 449F/H/V, 451T, 452S, 453A/L) among a large set of therapy naïve patients whose viruses did not carry primary resistance mutations (n = 1846). While polymorphisms were relatively frequent, in particular at position 449 and 451, the residues involved were clearly distinct from therapy-associated mutations. Only three therapy-associated mutations (436R, 453L and 437V) were found in more than 1% of these patients (8.5%, 5.7% and 1.7%, respectively). In striking contrast, in a group of therapy naïve patients who carried primary PIs resistance mutations (suggesting transmission of a resistant virus) the 431V mutation was found at a high frequency (22%), while this mutation did not exceed 0.5% in patients from the first group [12]. As in most other studies, the large majority of samples were from subtype-B HIV-infected patients. Differences in the prevalence of mutations and polymorphisms were noted between subtype-B and non-B viruses, although the limited number of samples from each subtype did not allow specific association. This is a relevant issue, since polymorphisms in Gag may facilitate the selection of specific protease mutations, by reducing their fitness cost.

The frequency and the kinetics of emergence of Gag cleavage site mutations during the course of evolution of HIV resistance to PIs have not been fully evaluated. Several studies have compared treatment-naïve and -experienced patients, and concluded on a higher frequency of mutations in the second group. However, to quantify the difference, relatively large cohorts should be considered, and group definition should be clearly stated. Verheyen *et al*. [37] reported that approximately 10% of treatment-naïve patients without primary resistance mutations (n = 275) harbored treatment-associated mutation(s) in NC/SP2/P6 cleavage sites. In the same study, 60% of treatment-experienced patients carrying at least one primary resistance mutation (n = 225) had one or more cleavage site mutation [37]. The frequency appears to increase with the duration of exposure to PIs, since highly evolved viruses from patients having failed multiple lines of PI-based antiretroviral therapy, carrying multiple resistance mutations in the protease and expressing high levels of resistance, almost always bear mutations in Gag cleavage sites [38]. It is worth stressing that subtype-B viruses were largely prevalent in these studies. The emergence of subtype-specific Gag mutations and their implication in resistance development deserve dedicated surveillance, as different solutions for resistance may characterize genetically distinct viruses. As an example, a recent study on subtype-G infected patients (n = 21) suggested that the frequent polymorphism 453T favored the selection of 453I rather than the treatment-associated 453L change observed in subtype-B viruses [49].

Because mutations in Gag were until recently essentially considered as fitness compensatory changes rather than as true resistance mutations, most resistance genotype assays do not take these mutations into account in their interpretation algorithms. These algorithms are for the most part based on correlations between response to PI treatment and genotype. Furthermore, if Gag cleavage site mutations emerge in the context of heavily mutated protease genes, their individual statistical impact on clinical response is likely to be confounded by the overall protease genotype. There are, however, some indications that the presence of Gag cleavage site mutations may correlate with virological outcome in patients in whom first line PI therapy has failed and in whom salvage therapy involves another compound of the same class [35,50]. This further suggests that these mutations not only affect viral fitness, but could also act as *bona fide* resistance mutations.
